# Transcatheter Occluder Devices for the Closure of Atrial Septal Defect in Children: How Safe and Effective Are They? A Systematic Review

**DOI:** 10.7759/cureus.25402

**Published:** 2022-05-27

**Authors:** Tejasvi Kashyap, Muhammad Sanusi, Elina S Momin, Asma A Khan, Vijayalakshmi Mannan, Muhammad Ahad Pervaiz, Aqsa Akram, Abeer O Elshaikh

**Affiliations:** 1 General Practice, California Institute of Behavioral Neurosciences & Psychology, Fairfield, USA; 2 Internal Medicine, California Institute of Behavioral Neurosciences & Psychology, Fairfield, USA; 3 Medicine, California Institute of Behavioral Neurosciences & Psychology, Fairfield, USA; 4 Urology, California Institute of Behavioral Neurosciences & Psychology, Fairfield, USA; 5 Internal medicine, California Institute of Behavioral Neurosciences & Psychology, Fairfield, USA

**Keywords:** pediatrics, children, efficacy, safety, adverse effects, complications, occluder devices, septal occluder devices, transcatheter occluder devices, atrial septal defect

## Abstract

Atrial septal defect (ASD) is a hole in the interatrial septum (IAS) of the heart that is one of the most common congenital heart diseases (CHD). Percutaneous transcatheter device occlusion is one of the techniques that have been developed for the closure of atrial septal defects. The primary objective of this study is to assess the safety and efficacy of septal occluder devices in the management of atrial septal defect in children. We searched PubMed, Science Direct, and Google Scholar databases to collect relevant articles according to a predetermined eligibility criteria and included 21 papers of different study designs in this systematic review. We found that transcatheter closure is safe and effective in most children with ASD. The major complications reported could be avoided by comprehensive clinical assessment and echocardiographic evaluation to determine appropriate device size and implantation strategy per individual child. Further research involving more clinical trials with larger sample size and longer duration of followup is required to improve the safety of existing devices for their use in *all* children with ASD despite their weight and defect size, and also the efficacy of newer devices such as biodegradable septal occluders.

## Introduction and background

Every year, about one to two out of 1,000 live babies are diagnosed with atrial septal defect [1], which is the second most common congenital heart disease (CHD). Atrial septal defects (ASD) make up 10%-15% of all congenital heart diseases [1].

During the fourth week of gestation, atrial septa grow caudally as the septum primum and septum secundum from the roof of the atria, dividing them into the right and left atria [2]. The two atria communicate during fetal life through a space between the septum primum and septum secundum called the foramen ovale [2]. The two septa normally fuse as a single septum soon after birth, serving as a barrier between the right and left atria [2]. A hole in this septum is known as an atrial septal defect. There are four types of atrial septal defects depending on the location: ostium secundum defect, ostium primum defect, sinus venous defect (further classified as superior and inferior), and coronary sinus defect [2]. Among them, ostium secundum defect is the most common [2].

**Figure 1:**
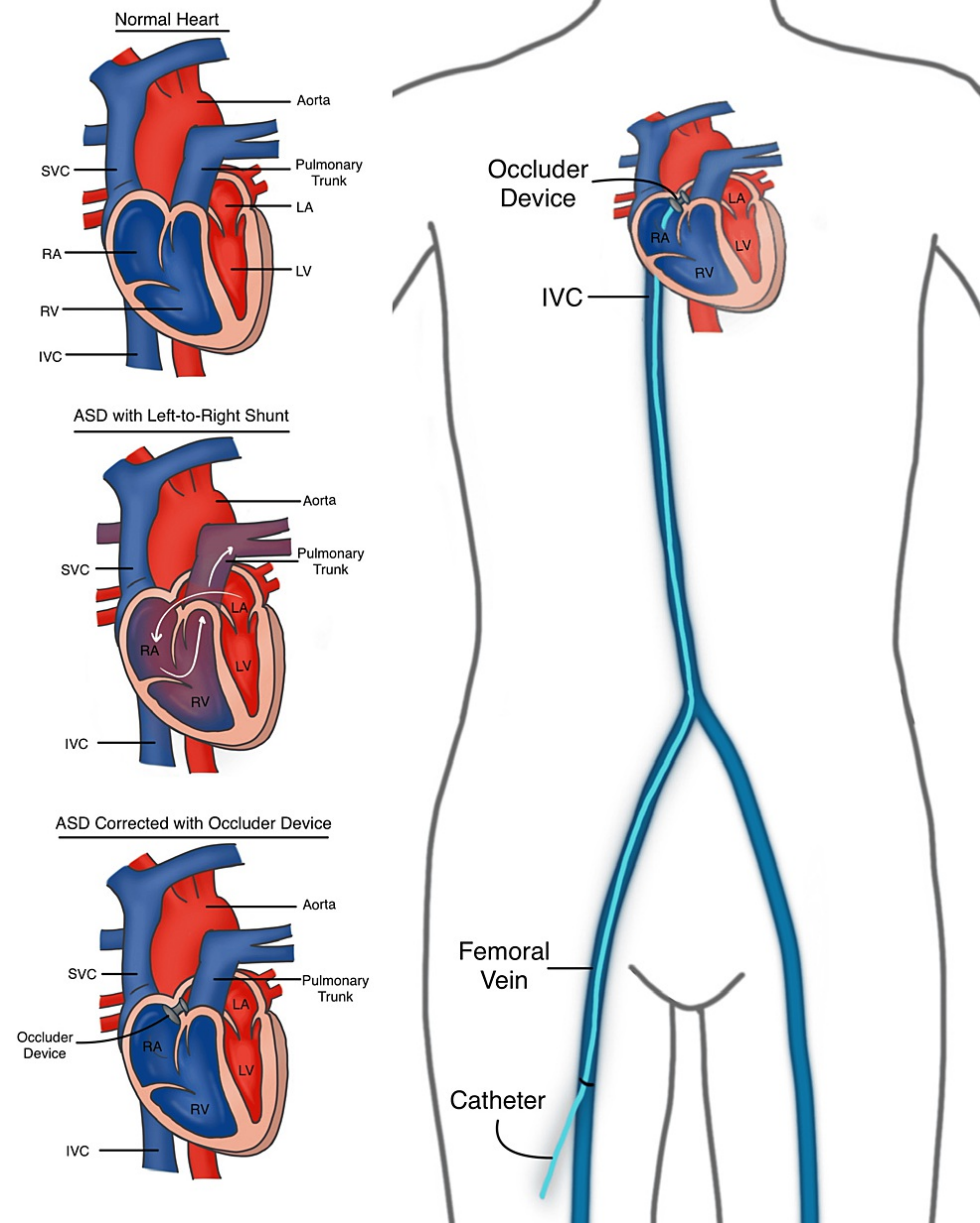
Transcatheter device closure of atrial septal defect (ASD) SVC - Superior Vena Cava, IVC - Inferior Vena Cava; RA - Right Atrium; RV - Right Ventricle; LA - Left Atrium; LV - Left Ventricle This figure is an original diagram created by the first author of the paper (Tejasvi Kashyap) using Procreate for the iPad.

Atrial septal defect serves as a window between the two atria that should not exist after birth. It is usually an acyanotic congenital heart disease, with a shunt of blood flowing from the left atrium to the right atrium as shown in Figure 1 [2]. Patients are usually asymptomatic and often undiagnosed till adulthood [2]. Large defects can present with exercise intolerance, arrhythmias, pulmonary hypertension, increased incidence of pneumonia, and increased mortality [2]. There is also a possibility of reversal of the shunt with blood flowing from the right atrium to the left atrium, known as Eisenmenger Syndrome, when right atrial pressures exceed that of the left, leading to cyanosis, dyspnea on exertion, increased pulmonary vascular resistance and increased susceptibility to infection [2]. Another serious potential complication of atrial septal defect is transient ischemic attack/stroke [2].

Spontaneous closure of ASD in the first year of life commonly occurs in patients with ASD smaller than 5mm [2]. Defects larger than 1cm usually require medical or surgical closure [2]. Previously, surgical closure was the standard of care for ASD, but over the last 40+ years, transcatheter devices have rapidly emerged as the routine in children [3,4]. As of today, the devices currently available for ASD closure include the Amplatzer Septal Occluder, Occlutech Figulla Flex II, Gore Cardioform Septal Occluder, Cocoon Septal Occluder, CeraFlex, Nit Occluder ASD-R, CardiO-Fix Septal Occluder, Ultracept II ASD Occluder, and Carag Bioresorbable Septal Occluder [5]. 

In transcatheter device closure of ASD, a catheter enclosing the septal occluder device is inserted through a vein in the groin (right femoral vein) under echocardiographic (transesophageal echocardiography/TEE) and/or fluoroscopic guidance and traversed upwards through the inferior vena cava (IVC) to the atrial septal defect as illustrated in Figure 1 [6,7]. The occluder device within the catheter exists folded up as an umbrella, and is pushed through the catheter to plug the defect in the atrial septum, after which the catheter is removed [4]. Eventually, cardiac tissue grows over the device (endothelialization), further securing it in place [6]. Unlike surgical closure of ASD, transcatheter device closure has a short post-operative recovery time, and requires no incision [8].

Prior to device closure of ASD, patients must be assessed for hemodynamics (in patients with right-to-left shunt), morphologic characteristics of the defect (size and presence of sufficient rim), presence of multiple defects, and presence of other cardiac conditions/abnormalities [5,9]. Large defects may lead to prolapse of the left atrial disk of the device into the right atrium [5]. Large defects with rim deficiencies may lead to further complications such as device embolization, impingement of nearby cardiac structures, and erosion [9]. Transcatheter device closure of ASD is indicated in children with acceptable hemodynamics with suitable anatomical features, transient right to left shunt with history of paradoxical emboli, right to left shunt with symptomatic cyanosis who do not require the communication to maintain cardiac output, and small ASDs with suspected high risk of thromboembolic events [9,10]. It is not indicated in patients with ASD other than septum secundum defect, small septum secundum defects without hemodynamically significant shunt and other risk factors, and in patients with advanced pulmonary vascular obstructive disease [10].

This systematic review aims to assess the safety and efficacy of septal occluder devices in the management of atrial septal defect in children. Patent foramen ovale (and other congenital heart diseases) were not included in this study.

## Review

Methods

Study Protocol

We created a systematic review in accordance with the Preferred Reporting Items for Systematic Review and Meta-Analyses (PRISMA) 2020 Guidelines [11].

Sources of Data Collection and Search Strategy

We reviewed scientific literature from three databases, PubMed, Science Direct, and Google Scholar, using keywords with Boolean words and medical subject heading (MeSH) from the last five years (2017-2022). Our search strategy is detailed in Table 1.

**Table 1:** Search Strategy

DATABASE	SEARCH	RESULTS
PUBMED	Atrial Septal Defect, and Septal Occluders. In PubMed, the final search strategy with keywords and MeSH used was as follows: (( "Heart Septal Defects, Atrial/complications"[Mesh] OR "Heart Septal Defects, Atrial/surgery"[Mesh] OR "Heart Septal Defects, Atrial/therapy"[Mesh] )) AND ( "Septal Occluder Device/adverse effects"[Mesh] OR "Septal Occluder Device/therapeutic use"[Mesh] )	20
GOOGLE SCHOLAR	atrial septal defect OR ASD AND Septal Occluder Device OR septal occluder OR occluder device OR occluder OR closure AND complications OR adverse effects OR safety AND children OR pediatric	100
SCIENCE DIRECT	atrial septal defect AND Septal Occluder Device OR septal occluder OR occluder device OR occluder OR closure AND complications OR adverse effects AND pediatric	100
Total		220

Eligibility Criteria

The inclusion and exclusion criteria for filtering papers are listed below in Table 2.

**Table 2:** Eligibility criteria

Inclusion Criteria	Exclusion Criteria
- Free full text	- Unavailable free full text
- Papers published in the last five years (from 2017-2022)	- Papers published before 2017
- Studies done in children (birth-18 years)	- Studies with results not specific to children with ASD
- Articles in English language only	- Articles not in English
- Worldwide	- Gray Literature
- All types of studies	- Duplicate studies

Data Extraction

We extracted data from the included studies and recorded them under the following headings: authors, year of publication, location, type of study, brand of atrial septal occluder device, sample size, age range, duration of follow-up, percentage of complete closure of the defect, sizes of ASD and occluder devices, status of septal rims, complications, intervention for complication, and associated factors in Table 3.

**Table 3:** Data extraction table

	Author	Year of publication	Location	Type of study	Device	Sample size	Age range	Follow-up	Complete Device Closure (efficacy)	ASD size	Occluder size	Aortic rim	Other rims	Complications (safety & efficacy)				Intervention	Associated factors/comments
															Intraprocedural	Post-op Follow-up	At last follow-up		
[12]	Yifan Li et al	2021	China	Clinical Trial	Absnow Biodegradable Occluder	n = 5	3.1-6.5 years	3 years	40% (⅖) Clinical closure 80% (⅘)	5-30mm	4-8mm larger than defect size sufficient rims: device 4-6mm larger than defect floppy rims/multiple defects: device 7-8mm larger than defect In general, PLLA device 2mm larger than metal device for same defect		Sufficient superior to mitral valve by 7mm superior to coronary sinus, SVC, IVC, & pulmonary vein by 5mm	Right atrial disk malformation	-	12 months	n = 1 (36 months)	-	
														Residual shunt > 4mm, RV enlargement	-	-			
														Residual shunt 2-4mm	-	-	n = 2 (36 months)	-	-
[13]	Jun-Yi Wan et al	2017	Taiwan	Case Report	Amplatzer Septal Occluder	n = 1	7 years	-	-	12mm	16mm	❌		Erosion → fistula	-	-	n = 1 (1 month)	Fistula closed w/ PDA occluder device (Lifetech Scientific)	-
[14]	Yasuko Onakatomi et al	2019	Japan	Case Report		n = 1	7 years	5 years	-	20mm	24mm	❌		Erosion → pericardial effusion, cardiac tamponade w/ collapsed RV, shock	-	-	n = 1 (5 years, 10 months)	Tear(s) repaired, device removed, ASD patch closure	-
[15]	Zai-Qiang Zhang et al	2021	China	Case Report		n = 1	16 years	-	-	6mm	12mm	❌	Sufficient 5mm from right pulmonary veins, SVC, os of coronary sinus, & mitral valve	Erosion/perforation → moderate pericardial effusion, hemopericardium, shock	-	-	n = 1 (3 months)	Tear(s) repaired, device removed, ASD patch closure	-
[16]	Wen-long Zhang et al	2021	China	Case Report		n = 1	5 years	-	-	15.2mm x 13.6mm	15mm	❌	Sufficient 5mm from right pulmonary veins, SVC, os of coronary sinus, & mitral valve	Erosion/perforation → pericardial effusion, hemopericardium, red thrombosis	-	-	n = 1 (1 month)	Tear(s) repaired, device removed, ASD patch closure	-
[17]	Bharti Sharma et al	2019	India	Retrospective Cohort Study		n = 45	8-38 months	1-36 months	95% (43/45)	-	equal to/ up to 10% more than ASD diameter	❌ in most cases	Sufficient SVC rim, IVC rim, posterior rim > 4mm AV valve/mitral rim > 7mm	Trauma to anterior mitral leaflet → Grade II MR	Grade II MR continued	-	n = 1 (18 months)	MV repaired	-
														Conduction block	n = 1 (Transient Mobitz Type I AV Block)	-	-	IV steroids, atropine	-
															-	n = 1 (2:1 AV block)	-	Oral steroids, NSAIDs	-
[18]	Mehdi Ghaderian et al	2019	Iran	Prospective Cohort Study		n = 35	6-14 months	29 months	77% (27/35)	≥6mm	equal to ASD diameter for ASD < 10mm 1-2mm > ASD diameter for ASD > 10mm	❌ (deficient in 28.3%; n = 321)	Sufficient	Arrhythmia	n = 2 (PSVT)	-	-	-	Resolved immediately without treatment
														Cerebral thrombosis → seizure, right sided hemiparesis	-	n = 1; (after 8 hours)	-	Treatment initiated immediately & symptoms corrected during follow-ups	ASD size larger & duration of procedure longer than other patients
[19]	Zakaria Jalal et al	2018	France	Retrospective Cohort Study		n = 1,326	0.7 - 18 years	6 months - 18 years	95.32% (1,264/1,326)	5-40mm by Echocardiogram 6-42mm by balloon sizing	4-40mm	❌ (deficient in 28.3%; n = 321)	Rim deficiencies: posterior - 14.2% (n = 161) anteroinferior - 9.8% (112) posterosuperior - 2.2% (n = 25) inferior - 13.6% (n = 155) superior - 4.3% (n = 49)	Device embolization	n = 7	n = 10	-	Device removed	-
														Unstable device	n = 5	-	-	Device removed	-
														AV valve damage	n = 2	-	-	Device removed	-
														Conduction block	n = 2 (reversible AVB)	-	-	Device removed	-
															-	n = 5 (2 CAVB, 2 AVB II, 1 AVB 1)	-	Device removed (n = 2; one patient with CAVB, and another patient with persistent asymptomatic suprahisian AVB II) Remaining (n = 3) resolved spontaneously/with systemic corticosteroids	-
														Trivial residual shunts	n = 47	-	-	-	-
														Arrhythmias	-	n = 8	n = 3	Anti arrhythmic drugs (n = 5)/catheter ablation (n = 2)/electrical cardioversion (for AF, n = 1)	-
														Pulmonary hypertension	-	n = 2 (after 1 month)	-	-	-
														Transient ischemic stroke	-	n = 2 (after 3 months)	-	Antiplatelet therapy	Occurred while receiving anti platelet therapy; no thrombus
														Migraine/headache	-	n = 15	-	-	-
														Atypical chest pain	-	n = 3	-	-	-
[20]	Han-Fan-Qiu et al	2019	China	Retrospective Cohort Study	Amplatzer Septal Occluder & Domestic ASD Device (Shanghai Shape Memory Alloy Co., Ltd., Shanghai, China; modified from Amplatzer ASD occluder)	n = 45	2-7 years	12-15 months	100%	-	1-2mm > ASD diameter	-	Sufficient	Device embolization	-	n = 1	-	Device removed, surgical ASD closure	-
														Arrhythmias	-	n = 11	-	-	Transient
														Hematoma at access site	-	n = 3	-	-	-
[8]	Yangyang Han et al	2020	China	Retrospective Cohort Study	Domestic ASD Device (Shanghai Shape Memory Alloy Co., Ltd., Shanghai, China; modified from Amplatzer ASD occluder)	n = 88	0-3 years	26-86 months	94.31% (83/88)	-	-	-	Sufficient ≥5mm distance from defect edge to coronary sinus, SVC, IVC, pulmonary vein 7mm distance from defect edge to AV flap	Device embolization	n = 1	-	-	Device removed, surgical ASD closure	Device embolization → cardiac arrest & blood flow interruption → minor brain complication → improved after treatment
														Oblique position of device on relatively large defect	n = 1	-	-	Device removed, surgical ASD closure one week later	-
														Unstable device	n = 3	-	-	Reimplantation with larger device	-
														Arrhythmias	n = 8 [+ AVB I (n = 3); + AF (n = 5)]	-	-	-	Transient
[21]	Priya Pradhan et al	2021	India	Retrospective Cohort Study	Amplatzer Cribriform Septal Occluder	n = 16	2.5-10.5	1-60 months	68.75% (11/16)	Multiple fenestrations ≥3 with major defect <12mm	≥1.5 x FSL (fenestrated septal length) but < TSL (total septal length)	-	Sufficient rim ≥4mm from defect to SVC/IVC, coronary sinus, mitral valve, RUPV	Residual shunt < 3mm	-	-	n = 3	-	-
[22]	Basil (Vasilios) D. Thanopoulos et al	2021	Greece	Retrospective Cohort Study	Cocoon Septal Occluder	n = 1853	2-14 years	12 - 84 months	99.4% (1800/1853)	-	equal to ASD diameter	❌ in 5.1% (n = 95)	Sufficient	Device embolization	n = 8	-	-	Device removed	-
														Conduction block	n = 16 (15 AVB I & II, 1 CAVB)	-	-	-	-
														Arrhythmias	n = 31 (atrial)	-	n = 38 (atrial)	-	-
														Migraine/headache	-	n = 25; (after 1-2 weeks)	-	Acetaminophen	-
[23]	Hyam Mahmoud et al	2019	Romania	Prospective Cohort Study		n = 27	3-25 years	3-26 months	88.9% (24/27)	8-26mm by TEE 13.5-32mm by balloon sizing	8-32mm	❌	Sufficient patients w/ deficient inferior/superior/posterior rims were excluded, especially if aortic rim also deficient	Device embolization	-	n = 2; (after 12 hours)	-	Device removed	Patients had < 5mm deficient posterior rim
														AV femoral fistula	-	n = 2	-	-	Disappeared spontaneously
														Hematoma at access site	-	n = 2	-	-	-
[24]	Amal M. El-Sisi et al	2021	Egypt	Retrospective Cohort Study	Occlutech Accel Flex II Septal Occluder	n = 30	5-18 years	5 years	100%	12-30mm	2-4mm larger than largest ASD diameter (10-33mm)	-	-	Sinus tachycardia	n = 2		-	-	-
														Mild MR	n = 2			-	-
														Mild AR	n = 1			-	-
														Mild TR	-	-	n = 11	-	-
														Mild PR	-	-	n = 5	-	-
[25]	Murat Muhtar Yilmazer et al	2018	Turkey	Prospective Cohort Study	Solysafe Septal Occluder	n = 25	5-12 years	5.2-7.2 years	88% (22/25)	6-21mm by TTE 7-23mm by TEE 8-26mm by balloon catheter	15mm for 4-12mm defects (n = 9) 20mm for 13-17mm defects (n = 8) → procedure failure in one 25mm for 18-22mm defects (n = 6) 35mm for 27-30mm defects (n = 2) → procedure failure in both	-	Sufficient > 5mm inferior & superior rims	Failure of device deployment	n = 3 (1 aneurysmal floppy septum, 2 floppy rims)	-	-	Device removed	Device embolization (n = 1)
														Residual shunt	-	-	n = 1 (6 years)	-	-
														Wire fraction	-	-	n = 1 (6 years)	-	-
														Left hemispheric infarct → right hemiparesis	n = 1	-	-	Physical therapy	-
														Arrhythmia	n = 1 (junctional rhythm)	-	-	-	Spontaneous resolution
														Partial occlusion of right femoral vein	n = 1	-	-	Heparin infusion → resolution	-
[26]	Gustaf Tangho¨j et al	2017	Sweden	Retrospective Cohort Study	Amplatzer (n = 212) Gore occluder (n = 20) Cardioseal (n= 4) Occlutech Figulla Flex (n = 7 Cocoon/vascular innovations (n = 8) Cardia atriasept (n = 1)	n = 252	0 - 18 years	-	-	5-21mm in children <15kg 4-21mm in children >15kg	6-33mm in children <15kg 5-36mm in children >15kg	-	-	Fatal device erosion	-	n = 1; (after 5 days)	-	-	Emergent surgical procedure required in 4 patients
														Arrhythmias	n = 7 (2 major, 4 minor, 1 prolonged)	-	-	Major arrhythmias required treatment	
														Pulmonary hypertension crisis	n = 1	-	-	-	
														Hypotension	n = 2	-	-	1 required treatment	
														Bleeding	n = 3	-	-	2 required transfusion	
[27]	Seul Gi Cha et al	2021	Korea	Retrospective Cohort Study	Amplatzer Septal Occluder (n = 280) Amplatzer Cribriform Septal Occluder (n = 2) Amplatzer PFO Occluder (n = 1) Cocoon Septal Occluder (n = 36) Occlutech Figulla Flex II (n = 81) Gore Cardioform Septal Occluder (n = 1)	n = 407	2-5 years	3.6-140.8 months	86.7% (353/407)	-	1-2mm > ASD diameter in TEE 0-1mm < balloon diameter in TEE	❌	MV rim > 5mm IVC rim 3-5mm cutoff SVC, PS, PI rims 1-3mm cutoff No multiple rim deficiency	Device deployment failure	n = 4	-	-	-	Failure
														MV problem	n = 3 (MV compression)	-	-	-	Failure
															-	n = 1 (LA disk touching MV)	-	-	-
														Conduction block	n = 1 (CAVB)	-	-	-	Failure
															-	n = 1 (CAVB)	-	-	-
														Device embolization	-	n = 1	-	Device removal & reimplantation of larger device - successful	-
														RV failure	-	n = 1	-	Device removed	-
														Misdiagnosis of PAPVR	-	n = 1	-	Device removed	-
														Aggravation of MR	-	-	n = 5	-	-
														Device leakage	-	-	n = 44	-	-
[28]	Safaa H. Ali et al	2017	Egypt	Retrospective Cohort Study	Amplatzer Septal Occluder, cribriform ASD occluder and delivery system (n = 132) Figulla-Occlutech device (n = 3)	n = 135		2 years	98.5%	-	20-25% larger than ASD diameter 20% if all rims preserved (except retro-aortic) 25% if 2 rims deficient equal to or 2mm larger than ASD diameter in children < 5 years	❌	Sufficient > 5mm distance between defect edge to mitral & tricuspid valves, SVC, RUPV, & coronary ​​sinus	Device embolization	n = 1	-	-	Device removed, surgical ASD closure	-
														Hemopericardium, cardiac tamponade	-	n = 2 (1 with erosion, 1 without erosion)	-	Pericardiocentesis, device removed (for patient with erosion)	-
														Conduction block	n = 2 (1 complete heart block, 1 II degree heart block)	-	-	Oral steroids → resolved in 2 days	Patient with II degree heart block had Down’s syndrome, IAS aneurysm, & multiple fenestrations closed by Cribriform device
														Arrhythmias	n = 2	-	-	Spontaneous resolution/resolution with catheter manipulation	Transient
														Rebleeding from access site	n = 1	-	-	-	-
														Residual shunt 3mm	-	n = 1	-	-	-
[29]	Mateusz T. Knop et al	2018	Poland	Prospective Cohort Study	Amplatzer Septal Occluder (n = 145) Amplatzer Cribriform Septal Occluder (n = 2) Cardi-O-Fix ASD Occluder (n = 2	n = 157	0-3 years	0.1-14.7 years	94.9% (49/157)	-	20-30% larger than ASD diameter in centrally located defects an stable atrial septums	❌ residual/absent	Sufficient ≥5mm excluding patients with residual/absent aortic rims accompanied by another floppy rim	Arrhythmias	n = 1 (major, SVT)	-	-	Long-term anti arrhythmic therapy for 2 years	-
															n = 4 [minor; SVT (n = 2), extrasystole (n = 2)]	-	-	SVT - adenosine bolus (n = 1); spontaneous resolution (n = 1)	-
														Conduction block	n = 1 (AVB II)	-	-	Steroids → resolved	-
														Respiratory tract infection	-	n = 5	-	-	-
														Anemia	-	n = 1	-	Transfusion	-
														Mitral valve insufficiency	-	-	n = 2	-	-
[30]	S. Ackermann et al	2018	Switzerland	Retrospective Cohort Study	Amplatzer Septal Occluder (n = 312 Solysafe Septal Occluder (n = 45) CeraFlex ASD Occluder (n = 11) Gore Cardioform Septal Occluder (n = 13) pfm NitOcclud ASD-R-Device (n = 18) BioSTAR Device (n = 8) HELEX Septal Occluder (n = 3)	n = 397	3.8-10.6 years	1 year	96.47% (383/397)	mean: 12.3-13.5mm	mean: 13.6-15.1mm	❌ sufficient rims ≥5mm seen in n = 191 short/deficient rims seen in n = 160		Equipment failure	n = 1	-	-	-	-
														Device embolization	n = 3	n = 2 (within 24 hours)	-	Device removed	Seen in Amplatzer Septal Occluder in patient with large defect >18mm
														Arrhythmias	n = 6	n = 4 (within 24 hours)	-	-	-
														Pericardial effusion	-	n = 2 (within 24 hours)	-	-	-
														Vascular access problems	-	n = 1 (within 24 hours)	-	-	-
														Impairment of neighboring cardiac structures	-	n = 3 (within 24 hours)	-	-	-
														Erosion	-	n = 2 (within 30 days)	-	-	+ fistula (n = 1)
														Thrombus formation	-	n = 3 (within 30 days)	-	-	-
														Mild MR	-	n = 1 (within 30 days)	-	-	-
														Conduction block	-	n = 1 (within 30 days; transient AV block)	-	-	-
														Residual shunt	n = 54 (16.5%; after 3 months)	-	(12% at one year)	-	Not hemodynamically significant
[31]	Jacinta Ng et al	2019	Australia	Case Report	Not specified	n = 1	18 years	-	-	-	-	-	-	Corynebacterium diphtheria-infective endocarditis	-	-	n = 1 (9 years)	Antibiotics → symptoms resolved; warfarin for possible thrombus	-

Risk and Quality Assessment

The articles were separately screened by two reviewers (T.K. and M.S.) using various quality appraisal tools including Joanna Briggs Institute (JBI) checklist for case reports and cohort studies, Cochrane bias assessment tool for randomized control trial, and Robin’s checklist for non-randomized control trial.

Results

**Figure 2:**
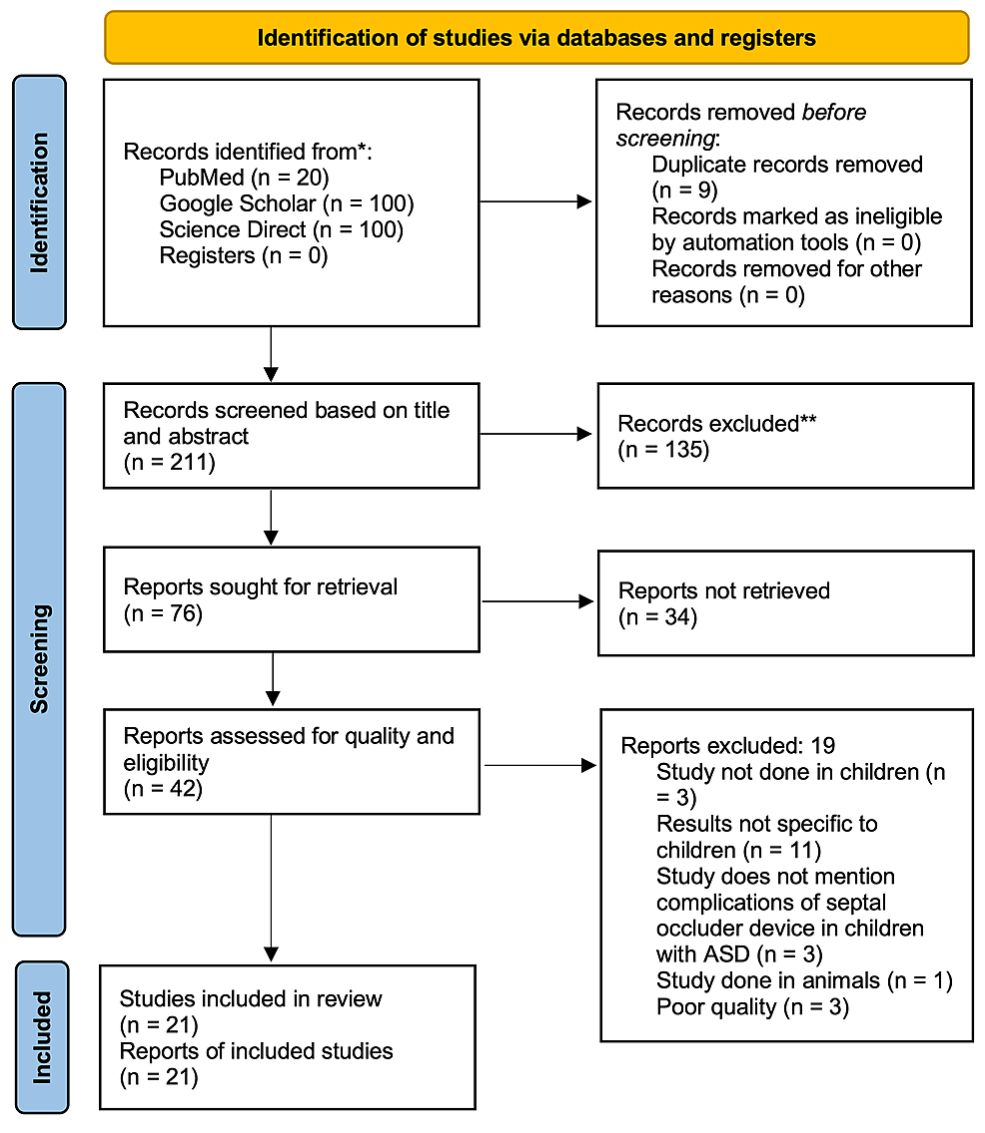
PRISMA 2020 flow diagram for new systematic reviews which included searches of databases, registers, and other sources

A total of 270,034 articles were identified after applying our search strategies: 251 from PubMed, 3,660 from Google Scholar, and 266,123 from Science Direct. A total of 220 articles remained after applying filters based on inclusion/exclusion criteria (PubMed), availability of free full text (PubMed, Science Direct), year of publication between 2017 and 2022, and including only the first 100 articles each from Google Scholar and Science Direct. Nine duplicates were found and deleted. 76 articles remained after screening based on the title and abstract, out of which 34 were excluded due to unavailability of free full text. The remaining 42 reports were assessed for quality and eligibility, leaving 21 articles total included in the review. PRISMA flow diagram is provided below in Figure 2.

Discussion

ASD closure is indicated in symptomatic children with recurrent respiratory tract diseases or failure to thrive requiring respiratory support at an earlier age, and >2 years of age in asymptomatic children weighing > 15kg [18]. Transcatheter device closure of young children requires more experience and skill [18]. According to the hemodynamics, defects in patients with ratio of pulmonary blood flow to systemic blood flow (Qp/Qs) >1.5 and/or dilated right atrium and ventricle are advised to be closed [8,18,24,25,29]. According to morphological features, patients must have adequate rims on echocardiographic evaluations [18].

Safety of septal occluder devices

The criterion for safety is the absence of serious adverse effects or device embolization during the follow-up period [12]. Our study found that the complications of percutaneous transcatheter device closure of ASD in children included device embolization, cardiac erosion or perforation leading to fistulas or pericardial effusion with or without cardiac tamponade, thrombosis, bleeding, valvular damage or regurgitation, arrhythmias, conduction blocks, and migraine, in various ASD occluder devices such as the Amplatzer, Cocoon, Occlutech, Solysafe, and Gore Cardioform Septal Occluders. The complications per study and associated factors are enumerated in Table 3. An overview of the complications and percentage of sample size with the complications is provided in Table 4.

**Table 4:** Complications of septal occluder devices MR = mitral valve regurgitation, AR = aortic valve regurgitation, PR = pulmonic valve regurgitation, TR = tricuspid valve regurgitation, AV = atrioventricular, TIA = transient ischemic attack

	Author	Device embolization	Cardiac erosion/perforation	Pericardial effusion	Hemopericardium	Cardiac Tamponade	Fistula	Mitral valve damage/compression/contact	MR	AR	PR	TR	AV valve damage	Thrombosis	TIA	Hemiparesis	Arrhythmias	Conduction Block	Vascular access site problems	Migraine/Headache	Infective endocarditis	Symptomatic patients	Sample Size	Occluder Device
[12]	Yifan Li et al	-	-	-	-	-	-	-	-	-	-	-	-	-	-	-	-	-	-	-	-	n = 0	n = 5	Absnow Biodegradable Septal Occluder
[13]	Jun-Yi Wan et al	-	✔️	-	-	-	✔️	-	-	-	-	-	-	-	-	-	-	-	-	-	-	n = 1	n= 1	Amplatzer Septal Occluder
[14]	Yasuko Onakatomi et al	-	✔️	✔️	-	✔️	-	-	-	-	-	-	-	-	-	-	-	-	-	-	-	n = 1	n = 1	
[15]	Zai-Qiang Zhang et al	-	✔️	✔️	✔️	-	-	-	-	-	-	-	-	-	-	-	-	-	-	-	-	n = 1	n = 1	
[16]	Wen-long Zhang et al	-	✔️	✔️	✔️	-	-	-	-	-	-	-	-	✔️	-	-	-	-	-	-	-	n = 1	n = 1	
[17]	Bharti Sharma et al	-	-	-	-	-	-	✔️	✔️	-	-	-	-	-	-	-	-	-	-	-	-	n = 1	n = 45	
		-	-	-	-	-	-	-	-	-	-	-	-	-	-	-	-	✔️	-	-	-	n = 2		
[18]	Mehdi Ghaderian et al	-	-	-	-	-	-	-	-	-	-	-	-	-	-	-	✔️	-	-	-	-	n = 2	n = 35	
		-	-	-	-	-	-	-	-	-	-	-	-	✔️	✔️	✔️	-	-	-	-	-	n = 1		
[19]	Zakaria Jalal et al	✔️	-	-	-	-	-	-	-	-	-	-	-	-	-	-	-	-	-	-	-	n = 17	n = 1,326	
		-	-	-	-	-	-	-	-	-	-	-	✔️	-	-	-	-	-	-	-	-	n = 2		
		-	-	-	-	-	-	-	-	-	-	-	-	-	✔️	-	-	-	-	-	-	n = 2		
		-	-	-	-	-	-	-	-	-	-	-	-	-	-	-	✔️					n = 8		
		-	-	-	-	-	-	-	-	-	-	-	-	-	-	-	-	✔️	-	-	-	n = 5		
		-	-	-	-	-	-	-	-	-	-	-	-	-	-	-	-	-	-	✔️	-	n = 15		
[20]	Han-Fan-Qiu et al	✔️	-	-	-		-	-	-	-	-	-	-	-	-	-	-	-	-	-	-	n = 1	n = 45	Amplatzer Septal Occluder & Domestic ASD device modified from Amplatzer
		-	-	-	-	-	-	-	-	-	-	-	-	-	-	-	✔️	-	-	-	-	n = 11		
		-	-	-	-	-	-	-	-	-	-	-	-	-	-	-	-	-	✔️	-	-	n = 3		
[8]	Yangyang Han et al	✔️	-	-	-	-	-	-	-	-	-	-	-	-	-	-	-	-	-	-	-	n = 1	n = 88	Domestic ASD device modified from Amplatzer
		-	-	-	-	-	-	-	-	-	-	-	-	-	-	-	✔️	-	-	-	-	n = 5		
		-	-	-	-	-	-	-	-	-	-	-	-	-	-	-	✔️	✔️		-	-	n = 3		
		-	-	-	-	-	-	-	-	-	-	-	-	-	-	-	-	-	✔️	-	-	n = 3		
[21]	Priya Pradhan et al	-	-	-	-	-	-	-	-	-	-	-	-	-	-	-	-		-	-	-	n = 0	n = 16	Cribriform Amplatzer Multifenestrated Septal Occluder
[22]	Basil (Vasilios) D. Thanopoulos et al	✔️	-	-	-	-	-	-	-	-	-	-	-	-	-	-	-	-	-	-	-	n = 8	n = 1,853	Cocoon Septal Occluder
		-	-	-	-	-	-	-	-	-	-	-	-	-	-	-	✔️	-	-	-	-	n = 38		
		-	-	-	-	-	-	-	-	-	-	-	-	-	-	-	-	✔️	-	-	-	n = 16		
		-	-	-	-	-	-	-	-	-	-	-	-	-	-	-	-	-	-	✔️	-	n = 25		
[23]	Hyam Mahmoud et al	✔️	-	-	-	-	-	-	-		-	-	-	-	-	-	-	-	-	-	-	n = 2	n = 27	
		-	-	-	-	-	-	-	-	-	-	-	-	-	-	-	-	-	✔️	-	-	n = 4		
[24]	Amal M. El-Sisi et al	-	-	-	-	-	-	-	✔️	-	-	-	-	-	-	-	-	-	-	-	-	n = 2	n = 30	Occlutech Accel Flex II Septal Occluder
		-	-	-	-	-	-	-	-	✔️	-	-	-	-	-	-	-	-	-	-	-	n = 1		
		-	-	-	-	-	-	-	-	-	✔️	-	-	-	-	-	-	-	-	-	-	n = 5		
		-	-	-	-	-	-	-	-	-		✔️	-	-	-	-	-	-	-	-	-	n = 11		
[25]	Murat Muhtar Yilmazer et al	✔️	-	-	-	-	-	-	-	-	-	-	-	-	-	-	-	-	-	-	-	n = 1	n = 25	Solysafe Septal Occluder
		-	-	-	-	-	-	-	-	-	-	-	-	-	✔️	✔️	-	-	-	-	-	n = 1		
		-	-	-	-	-	-	-	-	-	-	-	-	-	-	-	✔️	-	-	-	-	n = 1		
		-	-	-	-	-	-	-	-	-	-	-	-	-	-	-	-	-	✔️	-	-	n = 1		
[26]	Gustaf Tangho¨j et al	-	-	-	-	-	-	-	-	-	-	-	-	-	-	-	✔️	-	-	-	-	n = 7	n = 252	Nonspecific
[27]	Seul Gi Cha et al	✔️	-	-	-	-	-		-	-	-	-	-	-	-	-	-	-	-	-	-	n = 1	n = 407	
		-	-	-	-	-	-	✔️	-	-	-	-	-	-	-	-	-	-	-	-	--	n = 4		
		-	-	-	-	-	-		✔️	-	-	-	-	-	-	-	-	-	-	-	-	n = 5		
		-	-	-	-	-	-	-	-	-	-	-	-	-	-	-	-	✔️	-	-	-	n = 2		
[28]	Safaa H. Ali et al	✔️	-	-	-	-	-	-	-	-	-	-	-	-	-	-	-	-	-	-	-	n = 1	n = 135	
		-	✔️		✔️	✔️	-	-	-	-	-	-	-	-	-	-	-	-	-	-	-	n = 1		
		-	-	-	✔️	✔️	-	-	-	-	-	-	-	-	-	-	-	-	-	-	-	n = 1		
		-	-	-	-	-	-	-	-	-	-	-	-	-	-	-	✔️	-	-	-	-	n = 2		
		-	-	-	-	-	-	-	-	-	-	-	-	-	-	-	-	✔️	-	-	-	n = 2		
		--	-	-	-	-	-	-	-	-	-	-	-	-	-	-	-	-	✔️	-	-	n = 1		
[29]	Mateusz T. Knop et al	-	-	-	-	-	-	-	✔️	-	-	-	-	-	-	-	-	-	-	-	-	n = 2	n = 157	
		-	-	-	-	-	-	-	-	-	-	-	-	-	-	-	✔️	-	-	-	-	n = 5		
		-	-	-	-	-	-	-	-	-	-	-	-	-	-	-	-	✔️	-	-	-	n = 1		
[30]	S. Ackermann et al	✔️	-	-	-	-	-	-	-	-	-	-	-	-	-	-	-	-	-	-	-	n = 5	n = 397	
		-	✔️	-	-	-	-	-	-	-	-	-	-	-	-	-	-	-	-	-	-	n = 2		
		-	-	✔️	-	-	-	-	-	-	-	-	-	-	-	-	-	-	-	-	-	n = 2		
		-	-	-	-	-	-	-	✔️	-	-	-	-	-	-	-	-	-	-	-	-	n = 1		
		-	-	-	-	-	-	-	-	-	-	-	-	✔️	-	-	-	-	-	-	-	n = 3		
		-	-	-	-	-	-	-	-	-	-	-	-	-	-	-	✔️	-	-	-	-	n = 10		
		-	-	-	-	-	-	-	-	-	-	-	-	-	-	-	-	✔️	-	-	-	n = 1		
		-	-	-	-	-	-	-	-	-	-	-	-	-	-	-	-	-	✔️	-	-	n = 1		
[31]	Jacinta Ng et al	-	-	-	-	-	-	-	-	-	-	-	-	-	-	-	-	-	-	-	✔️	n = 1	n = 1	
	Total	n = 37	n = 7	n = 5	n = 4	n = 3	n = 1	n = 5	n = 10	n = 1	n = 1	n = 1	n = 2	n = 5	n = 4	n = 2	n = 92	n = 32	n = 13	n = 40	n = 1	n = 272	n = 4,848	-
	Percentage of total sample size	0.76%	0.14%	0.10%	0.08%	0.06%	0.02%	0.10%	0.20%	0.02%	0.02%	0.02%	0.04%	0.1%	0.06%	0.04%	1.8%	0.66%	0.26%	0.8%	0.02%	5.6%	-	-

Jalal et al.’s study found that children ≤15kg and children with large defects ≥20mm/m^2^ were more at risk for both periprocedural and long-term complications [19]. Tangho¨j et al.’s study also noted more complications in children <15kg than those >15kg [26]. Procedure-related challenges in young children <15kg include smaller sized vessels and atrial septums with increased difficulty in manipulation of catheters in the heart, ASD calibration with balloon catheter, oblique position of implant in ASD after opening the left atrial disk, and lack of patient-cooperation requiring longer sedation time [29]. Three of the studies included children with comorbidities such as other cardiac conditions, genetic abnormalities, and preterm births [26,29,30]. Knop et al.’s study even included children who underwent other interventions simultaneously along with ASD closure [29]. Tangho¨j et al.’s study found that 10% (n = 11) of children that weighed <15kg, 14% (n = 3) that had other cardiac comorbidities, 16% (n = 4) that had genetic abnormalities, 13% (n = 4) that had other comorbidities, and 7% (n = 3) that were born preterm had major complications, and none of them had minor complications except for 1% (n = 1) <15kg [26]. The same complications were seen in both children with comorbidities and those without, and presence of comorbidities does not seem to be associated with greater risk of complications.

Cardiac erosion

Among the listed complications, cardiac erosion/perforation was rare, but found to be the most notable and serious, particularly seen after deployment of Amplatzer Septal Occluder. Forty percent of cases of cardiac erosion are reported in children [19]. Possible risk factors for erosion include deficient rims, oversized device, impingement of atrial disks over aortic root, and extreme movement of device before release [13]. Most of the cardiac erosions occur near the aortic root and the top of the atrium [15]. After device closure of ASD, atrial sizes decrease as a result of the occluding device occupying space within it [16]. During each cardiac cycle, the septal occluder device (particularly if larger in size) can come in contact with and erode through the atrial roof and adjacent aorta [15]. Early erosion may only present with a small amount of pericardial effusion [16]. With growth of the child, the ratio of the device to the atrial septal diameter decreases, so late erosions/perforations were found to be rare [16].

Three case reports each depicted cardiac erosion in children 5-7 years of age with absent/deficient aortic rims, one month, five years, and one month respectively after transcatheter closure of ASD with Amplatzer Septal Occluder [13,14,16]. Zhang et al. also presented a case of an adolescent with cardiac erosion three months after the placement of Amplatzer Septal Occluder for closure of ASD with sufficient rims [15]. Among these four case reports of cardiac erosion, one patient subsequently developed a fistula from the aorta to the right atrium, and the other three had pericardial effusion and either hemopericardium or cardiac tamponade along with shock [13-16]. Ali et al. and Ackermann et al. both reported cardiac erosion in some of the children who underwent transcatheter closure of ASD using various brands of occluder devices [28,30]. In Ali et al.’s study, two of 135 patients developed hemopericardium and cardiac tamponade, among which only one had cardiac erosion [28]. In Ackermann et al.’s study of 397 children, two developed cardiac erosion, one of which led to the formation of a fistula (a similar finding to Wan et al.’s case report), three had impairment of neighboring cardiac structures, and two presented with pericardial effusion without cardiac erosion, all within 24 hours of occluder device employment [30]. Though the remaining cohort studies included in this review did not report cardiac erosion, it cannot be ruled out as a possible future complication due to the delay in its presentation, as cardiac erosion can occur up to even nine years after device closure of ASD, and some cases of erosion may also remain undetected and spontaneously resolve [19].

Oversized devices are sometimes chosen to close large defects with deficient aortic rims in order to cover the entire area of the defect [16]. Overinflation of the balloon during balloon-sizing of the atrial septal defect may also result in the selection of larger devices than necessary [30]. In two of the case reports of cardiac erosion, the occluder devices used were each 4mm larger than the defect, and in another case report, the device was double the size of the defect [13-15]. However, in Zhang et al.'s study, the child with deficient aortic rim was managed with a device equal in size to the defect and still developed aortic erosion, suggesting that absent aortic rim may be associated with higher risk of erosion than the use of an oversized device [16]. Thanopoulos et al.’s study that used Cocoon Septal Occluder for the transcatheter closure of ASD in 1,835 children did not exclude children with isolated rim deficiencies, and states that risks and complications related to the procedure can be avoided if the investigator selects an appropriate implantation strategy for each individual patient [22]. Their study followed up the children for 2-14 years and did not observe cardiac erosion or any other major complication [22].

Device embolization

Device embolization/occluder dislodgement is a rare but serious complication of device closure operations [20]. A total of 37 out of 4,278 children in eight of the studies experienced device embolization after transcatheter device closure of large defects or defects with insufficient septal rims either intra or postoperatively which had to be replaced or surgically removed with or without surgical patch closure of ASD [8, 19, 20, 22, 23, 25, 27, 28, 30]. The most cases of device embolization or migration were seen in Jalal et al.’s study [19]. Out of 1,326 children, 17 experienced device embolization - seven during the procedure and 10 post-operatively [19]. Their study also had the highest frequency of rim deficiencies compared to the other studies, which could be the major contributing factor for this complication [19]. Implant embolization could also be related to the operator’s learning curve [29]. Apart from device embolizations, Han et al.’s study also experienced intraprocedural issues such as unstable device in three patients, requiring reimplantation with larger devices, and oblique position of the device on a relatively large defect in one patient, requiring device removal and surgical closure of ASD one week later [8]. Unstable devices were also seen in five more patients in Jalal et al.’s study, leading to failure of implantation [19].

Thrombosis and transient ischemic attack/stroke

In Zhang et al.’s case report, a red thrombus was incidentally found attached to the anterior wall and root of aorta during intraoperative exploration after median sternotomy to remove the occluding device due to another complication (pericardial effusion and hemopericardium) [16]. Ackermann et al. also found three children with thrombus formation [30]. One of the 35 children in Ghaderian et al.’s cohort study had a cerebral thrombosis presenting with seizure about eight hours after closure of ASD with Amplatzer Septal Occluder, followed by right-sided hemiparesis [18]. This patient had a larger defect and a longer duration of stay than the other patients [18]. Right sided hemiparesis was also observed in another patient with left hemispheric infarct following implantation of a 15mm Solysafe Septal Occluder device into an 8mm defect in Yılmazer et al.’s cohort study of 25 children, and this patient was managed with physical therapy [25]. In Jalal et al.’s study, of 1,326 children who underwent transcatheter device closure of ASD using the Amplatzer Septal Occluder, two patients presented with transient ischemic stroke on anti-platelet therapy and without thrombus three months after transcatheter device closure of ASD [19].

Valve damage/regurgitation

Among 407 patients aged 2-5 years in a study by Cha et al. who underwent transcatheter device closure of ASD using various different brands of septal occluder devices, three patients had failure of closure due to compression of the mitral valve by the left atrial disk, one of which developed anterior mitral leaflet prolapse with mitral regurgitation, and the other two developed mild regurgitation [27]. In another patient, the left atrial disk of the device was touching the mitral valve [27]. Knop et al.’s study found mild mitral valve insufficiency in two of 157 patients under three years of age with secundum ASD who were managed with either Amplatzer Multi-Fenestrated Septal Occluder or Cardi-O-Fix ASD Occluder [29]. Patients in El-Sisi et al.’s study experienced the widest variety of valvular problems compared to the rest of the studies [24]. Among 30 patients aged 5-18 years who underwent transcatheter closure of secundum ASD using Occlutech ACCELL Flex II device, mild mitral and aortic regurgitation were seen in two children each that persisted till the last follow-up, at the time of which 11 more had mild tricuspid regurgitation and five more had mild pulmonary regurgitation [24]. Two of 62 children with occlusion failure in Jalal et al.’s study had failed due to atrioventricular valve damage, and one patient developed mild mitral regurgitation after successful device closure [19].

Arrhythmias and conduction abnormalities

Arrhythmias were found to be the most common minor complication among the included studies. Although ASD is known to alter atrial structure and depolarization, leading to increased risk of arrhythmias, transient arrhythmias such as supraventricular tachycardia, ectopy, and conduction abnormalities such as atrioventricular block have been seen after the closure of ASD [26]. Ghaderian et al. reported two cases of paroxysmal supraventricular tachycardia during placement of Amplatzer Septal Occluder device that resolved spontaneously without treatment [18]. One patient in Yılmazer et al.’s study developed junctional rhythm intra-procedurally after placement of Solysafe Septal Occluder which spontaneously resolved [25]. In Tangho¨j et al.’s study of 252 infants undergoing transcatheter closure of ASD using various brands of occluder devices, two developed major arrhythmias requiring treatment, one developed prolonged arrhythmia during the procedure, and four developed minor arrhythmias [26]. Out of 45 children undergoing transcatheter closure of ASD with Amplatzer Septal Occluder in Qiu et al.’s study, 11 developed transient arrhythmias/conduction abnormalities postoperatively, including first degree atrioventricular block (AVB I) and frequent ventricular premature beats in three patients, and atrial fibrillation in two patients [20]. Jalal et al.’s study also found two cases of intra-procedural reversible atrioventricular block, and five more cases of conduction abnormality after the procedure, among which two had complete AV block, two had AVB II, and one had AVB I [19]. three of them resolved either spontaneously or with systemic corticosteroids, and two patients required surgical device removal [19]. They also found eight cases of arrhythmias after the procedure, three of which persisted till the last follow-up [19]. Thanopoulos et al.’s study of 1,853 children undergoing device closure with Cocoon Septal Occluder noted 31 patients with atrial arrhythmias during the procedure which increased to 38 cases of minor atrial arrhythmias at six months of follow-up, and 16 cases of conduction block during the procedure, one of which was complete AV block, while the remaining were either first or second degree AVB (AVB I or II) [22]. Two more children in Cha et al.’s study developed complete AV block, one of which was during the procedure, leading to occlusion failure [27]. Sharma et al.’s study of 45 children weighing ≤10kg noted two cases of conduction abnormality after the deployment of Amplatzer Septal Occluder device [17]. One child developed a transient Mobitz Type I AV block immediately after placing the occluder device, which normalized after treatment with IV steroids and atropine [17]. The other child developed 2:1 AV block 24 hours after placing the occluder device, which normalized after treatment with oral steroids and NSAIDs [17].

Infective endocarditis

Ng et al. presented a case of Corynebacterium endocarditis in an 18-year-old patient nine years after uncomplicated implantation of a septal occluder device for ASD in Australia [31]. Echocardiography confirmed the presence of a vegetation at the left atrial surface [31]. ASD closure device-related endocarditis and Corynebacterium diphtheria endocarditis are both rare [31]. Seven of the 21 studies included in this review report that antibiotic prophylaxis was given to the children undergoing transcatheter closure of ASD, which was likely given to the children in the other studies as well, despite them not having reported it. None of them experienced infective endocarditis. It is unknown whether the child in Ng et al.’s study received antibiotic prophylaxis prior to or during the procedure [31].

Bleeding

Peripheral vascular injury hematoma can be seen commonly due to the requirement of peripheral vascular (femoral vein) approach for transcatheter mode of device closure of ASD [20]. Hematoma at the access site (groin) was reported in three patients in Qiu et al.’s study, two patients in Mahmoud et al.’s study, and three patients in Han et al.’s study, [8,20,23]. Ali et al.’s study had one patient with rebleeding at the access site [28]. One patient in Ackermann et al.’s study developed thrombosis of the right iliac artery [30]. Bleeding also occurred in three more patients in Tangho¨j et al.’s study, of which two were major and required transfusion [26]. Another patient in Knop et al.’s study required transfusion due to anemia [29]. Anemia was also seen in the patient with cardiac erosion and fistula in Wan et al.’s study [13].

Hypotension and pulmonary hypertension

In Tangho¨j et al.’s study, blood pressure drop was seen intraprocedurally in two patients, one of which required treatment, and pulmonary hypertension crisis was seen in one patient [26]. Pulmonary hypertension also was seen in two more patients in Jalal et al.’s study one month after device deployment [19].

Migraine/headache

Migraine was reported in two of the studies. In Thanopoulos et al.’s study, 25 children developed mild to moderate migraine within 1-2 weeks of Cocoon Septal Occluder implantation and were managed with oral medications [22]. Fifteen children in Jalal et al.’s study had migraine as a delayed complication after Amplatzer Septal Occluder implantation [19].

Comparison of different brands of septal occluders

As the Amplatzer Septal Occluder was the most commonly employed device for the closure of ASD, it presented with the widest range of complications overall. Complications seen with the use of Cocoon Septal Occluder included arrhythmias and conduction abnormalities, vascular access site problems, migraine, and even device embolization in two patients [22,23]. Children in one study using Occlutech Septal Occluder mostly experienced valvular regurgitation [24]. Four out of 25 children in one study using Solysafe Septal Occluder experienced device embolization, stroke leading to hemiparesis, arrhythmias, or vascular access site problems [25]. The remaining studies used various different septal occluders, the most common being the Amplatzer Septal Occluder, and displayed an array of complications.

Most of the major complications such as device embolization, or cardiac erosion/perforation and its accompanying complications are associated with larger sized septal defects with insufficient rims, particularly ≤4mm, and improperly sized devices. These can be avoided with careful assessment of anatomical and morphological features of the defect with appropriate selection of the device as well as method of deployment per individual if deemed adequate for transcatheter device closure. Increased experience and skill of the operating surgeon in performing percutaneous transcatheter device deployment could further reduce the risk of device embolizations and cardiac erosion. Determination of the size of atrial septal defect can be achieved with echocardiography alone, but if surgeons choose to perform balloon-sizing as well, they must exert caution during balloon inflation to ascertain not to oversize and subsequently choose a larger device for the defect. Mitral valve insufficiency was another observed complication that could be prevented with appropriate sizing of the occluder device. Arrhythmias and conduction abnormalities were the more frequently observed complications that more often than not resolved by the end of follow-up periods with or without treatment. Complications were also discovered more frequently in children <15kg due to their small size and lack of cooperation. Surgical closure of ASD should therefore be considered instead for children with larger defects with inadequate rims, as well as symptomatic children <15kg. Asymptomatic children with ASD are subject to reconsideration and may not require closure.

Efficacy of septal occluder devices

Complete Closure

Complete closure of ASD with the septal occluder devices was ≥ 94% in nine of the 21 studies at the end of their follow-ups [8,17,19,20,22,24,28-30]. The lower percentage of complete closure at last follow-up in the remaining studies is likely attributed to smaller sample size. Failure of complete closure of the septal defect was due to larger defect size. Further details are given in Table 3.

Residual Shunts

Residual shunts are common complications seen in device closure operations, especially in the presence of large defects [20]. Trivial or small residual shunts <2mm in size usually disappear during the follow-up period as endothelialization (tissue growth over the device) occurs, so they can be ignored [20]. Residual shunts detected by TTE are graded based on their size as mild (≤2.0mm), moderate (2.1-4.0mm), and large (≥4.0mm), and only large residual shunts with right ventricular enlargement are considered clinically significant [12]. Most of the studies included in this review that reported residual shunts were hemodynamically insignificant.

Device Leakage

Cha et al. reported 44 cases of device leakage at the end of 11 years of follow-up [27]. Compared to other studies who did not report such complications and only included children with septal rims of at least 4-5mm or more in size, Cha et al.’s study had a lower cutoff value for what they defined as acceptable rims [8,12,15,17,21,25,27,29].

Absnow Biodegradable Device

Li et al.’s study described the use of a biodegradable septal occluder, the Absnow device, in the closure of ASD [12]. One patient experienced right atrial disk malformation at 12 months of follow-up, which led to a large residual shunt >4mm and right ventricular enlargement by 36 months of follow-up, and was therefore considered a clinical closure failure [12]. Two more patients developed 2-4mm residual shunts at 36 months follow-up [12]. The device also had weak self-centrality and increased instability due to its soft material [12]. Biodegradable occluders are newer devices being developed which could potentially be safer than metal devices but require more research and improvement in order to reach the level of efficacy of the metal devices.

Amplatzer cribriform septal occluder for ASD with multiple fenestrations

Pradhan et al.’s study in India was exclusively done in children with multiple fenestrations (more than one defect) in the interatrial septum, so they employed the use of a single Amplatzer Cribriform Septal Occluder instead of separate devices per defect [21]. Sixteen children 2.5-10.5 years of age with adequate rims (>4mm) were included in the study [21]. The operators used a unique approach of passing the device through a small central fenestration rather than the major defect that allowed better device stability to the non-self-centering device [21]. Complete closure was seen in 11 of the 16 patients at the last follow-up, the remaining five of which had trivial residual shunts, and none of them experienced any complications, suggesting safety and effectiveness of both the device and their technique [21]. Cribriform occluders have been shown to be useful in patients with multiple fenestrations in the atrial septum and avoid the use of multiple devices to close the defects that lead to greater complications especially in children with smaller sized hearts and less space within the atria.

Limitations

Our study had a few limitations primarily related to brand of occluder device and sample size. Out of the 21 studies included in this review, ten were exclusively about the Amplatzer Septal Occluder (one of which was about a device modified from the Amplatzer Occluder, and another was specifically about the Cribriform Amplatzer Septal Occluder for fenestrated defects), as it is the most widely used device. Of the remaining studies included, two were exclusively about the Cocoon Septal Occluder, one of which had a small sample size. There was one study each exclusive to the Occlutech Accel Flex II and Solysafe Septal Occluder, both of which had small sample sizes. The results of the other studies were not specific to the different brands of septal occluder devices. Some of the studies also had patients lost to follow-up. Therefore, our review cannot be considered a fair representation of all the available septal occluder devices. Our study also excluded papers not written in the English language, as well as articles with unavailable free full text, so it is possible that many relevant articles with valuable information could have been omitted.

## Conclusions

In this systematic review, we assessed the safety and efficacy of various occluder devices. The currently available transcatheter septal occluder devices have been shown to be safe and effective in the closure of the atrial septal defect in most children, but further modifications and research in the form of clinical trials with large sample sizes of children from birth to 18 years of age, using all the available septal occluder devices (and documenting results specific to the brands), is still required in order to prevent the occurrence of major complications and ensure safety in every child with ASD, as well as improve efficacy in newly emerging biodegradable devices, and in the meantime, careful clinical and echocardiographic assessment of the children with ASD should be done in order to select the appropriate device and technique of implantation, and surgical repair should be considered instead for children with larger defects.
